# Disposable Nonenzymatic Uric Acid and Creatinine Sensors Using *μ*PAD Coupled with Screen-Printed Reduced Graphene Oxide-Gold Nanocomposites

**DOI:** 10.1155/2019/3457247

**Published:** 2019-02-03

**Authors:** Kamolwich Income, Nalin Ratnarathorn, Napassawan Khamchaiyo, Chanut Srisuvo, Leela Ruckthong, Wijitar Dungchai

**Affiliations:** ^1^Organic Synthesis, Electrochemistry & Natural Product Research Unit, Department of Chemistry, Faculty of Science, King Mongkut's University of Technology Thonburi, 126 Pracha-utid Road, Bang Mod, Thungkru, Bangkok 10140, Thailand; ^2^Department of Primary Industries and Mines, Ministry of Industry, Bangkok 10400, Thailand

## Abstract

Uric acid (UA) and creatinine are the imperative biological substance for clinical monitoring and diagnosis. Measuring the ratio between uric acid and creatinine in urine helps differentiate acute uric acid nephropathy from the hyperuricemia that secondarily occurs to renal failure. In general, the ratio is greater than 0.9 in acute uric acid nephropathy and less than 0.7 in hyperuricemia. In this work, disposable nonenzymatic screen-printed reduced graphene oxide-gold nanocomposites electrodes were firstly developed for the quantitative analysis of uric acid. Our sensors were also coupled with the paper-based colorimetric sensor of the determination of creatinine. Hence, an alternative high-throughput screening test for the uric acid to creatinine ratio with high sensitivity, specificity, simplicity, and rapidity was developed. Under the optimum conditions, our disposable nonenzymatic screen-printed electrode for the determination of uric acid shows the acceptable analytical performance in a wide range of linearity (2.5-1,000 *μ*M) with a low detection limit of 0.74 *μ*M. Our electrodes also showed no interference from common physiologic compound in urine. The determination of creatinine has been developed using Jaffé reaction between the creatinine and picric acid in alkaline condition. The alkaline picrate color on *μ*PAD changed from yellow to orange in the presence of creatinine and the orange intensity is directly proportional to the creatinine amount in a linearity range of 0.20-6.0 mM as a detection limit of 180 *μ*M. Finally, our device has been utilized to determine uric acid and creatinine simultaneously in control urine samples with acceptable result.

## 1. Introduction

Uric acid (UA) and creatinine are usual components in human fluids. The inordinate production of creatinine may result in an increase of* S*-adenosylmethionine degradation, ATP in creatinine, and purine metabolism, which subsequently prompts the synthesis of uric acid [1]. Uric acid level in urine corresponds to diseases and reflects the physiological state of human body [2]. Hypertension, cardiovascular disease, and gout have been linked to a level of uric acid in serum and urine over 420 *μ*M and 4.43 mM, respectively [3]. As well as with other conditions, having uric acid in serum and urine lower than 120 *μ*M and 1.48 mM may cause neurodegenerative diseases [4]. Therefore, average levels of uric acid in serum and urine for healthy human are between 120 to 420 *μ*M and 1.48 to 4.43 mM, respectively. Creatinine is the end product of creatine and phosphocreatine metabolism in muscles. Creatinine levels in serum and urine can be used as a biomarker for monitoring the deteriorated kidney function and the effect of treatment in hemodialysis patients [5]. Normal range of creatinine is between 40 to 150 *μ*M in serum and 2.48 to 22.92 mM in urine [6, 7]. If high uric acid levels in serum are persistently found, total uric acid excretion may be needed to estimate the kidney function by two 24-hour urine collections for monitoring uric acid excretion and creatinine clearance. The first 24-hour urine collection is performed while patients are on their usual diet and alcohol intake. In the second collection, the patient goes on a low-purine, alcohol-free diet for 6 days, with a repeat 24-hour urine collection performed on the last day. This method is a complex and time-consuming process, so an alternative rapid method for the measurement of uric acid to creatinine ratio still needs. The exploitation of uric acid to creatinine ratio in urine has lead in rapid monitoring the primary gout, the kidney disease states [8], and usefulness in the treatment of some diseases such as the hyperuricemia, renal failure, and acute uric acid nephropathy [9]. Chromatographic methods for the simultaneous determination of uric acid and creatinine were currently introduced such as high performance liquid chromatography [10], capillary zone electrophoresis [11], liquid chromatography-tandem mass spectrometry [12], and micellar electrokinetic chromatography [13]. Although these methods give high accuracy and sensitivity, they are quite complicated and require instrument. Hence, the development of an alternative method for the rapid, disposable, and simultaneous monitoring of uric acid and creatinine is still needed.

During the last decades, electrochemical technique has been announced as a possible method for the determination of uric acid due to its specificity, high sensitivity, rapidity, ease miniaturization, simple operation, and inexpensiveness [14, 15]. Many electrochemical sensors have been developed for the uric acid determination but the interferential species have effect on the uric acid electrochemical oxidation such as ascorbic acid (AA) and dopamine (DA) due to the electroactive nature of them [16, 17]. Herein, graphene nanomaterial has been modified on the electrode for the uric acid determination to overcome the low selectivity of electrochemical sensor. Graphene is beneficial as it is cheaper and stable to biodegradation than enzymatic system [18]. Jiao* et al. *established a nonenzymatic uric acid electrochemical sensor using graphene modified carbon fiber electrode [19]. Moreover, reduced graphene oxide modified with gold nanoparticles (AuNPs) onto glassy carbon electrode (GC) has been developed for uric acid sensor to improve some analytical performance. Most of recent electrochemical sensors for uric acid detection have been not invented on the disposable and portable electrode. The disposable and portable screen-printed carbon electrode (SPCE) offers the advantages over the bulk electrode including avoiding the issue of cleaning electrodes, renewable surface, and the reduction of cross infection. Furthermore, analysis can be done by single drop of sample onto electrode without the commonly used cumbersome electrodes and cells. Therefore, SPCEs modified with polydopamine functionalized reduced graphene oxide-gold nanocomposite (PDA-rGO/Au) have been proposed to achieve the highly sensitive, selective, portable and disposable nonenzymatic determination of uric acid. As well as, the simple Jaffé's colorimetric detection has been applied to evaluate the level of creatinine on *μ*PAD. The change in color of alkaline picrate from yellow to orange on the *μ*PAD due to the addition of creatinine was followed.

Our aim is to invent portable and disposable nonenzymatic sensors for the simultaneous determination of uric acid and creatinine with high sensitivity, specificity, rapidity, and simplicity. Our designed device can be a useful tool for practical applications in control urine samples.

## 2. Materials and Methods

### 2.1. Reagents and Chemicals

All chemicals used in experiment were of analytical reagent (AR) grade and solutions were prepared using deionized water, which was obtained from a Millipore Milli-Q water system. Sulfuric acid, nitric acid, hydrochloric acid, ethyl alcohol, hydrogen peroxide, potassium permanganate, sodium nitrate, sodium hydroxide, picric acid, and ammonium chloride were obtained from Merck. Ascorbic acid, calcium chloride, sodium hydrogen carbonate, sodium sulphate, citric acid, sodium hydrogen phosphate, disodium hydrogen phosphate, and sodium dihydrogen phosphate were received from BDH Prolabo. Potassium chloride and sodium chloride were obtained from Ajax Finechem Pty Ltd. Graphite (99.8%) and nafion (5%w/w) were purchased from the Fuel Cell Store. Tris(hydroxymethyl) aminomethane hydrochloride was obtained from Carlo Erba. Sodium borohydride was bought from Fluka Chemika. Hydrogen tetrachloroaurate trihydrate, dopamine hydrochloride, uric acid, creatinine, glucose, urea, trisodium citrate, and magnesium sulphate were obtained from Sigma Aldrich. Whatmam No.1 filter papers were purchased from Cole-Parmer (VernonHills, IL). The supporting electrolyte 0.1 M phosphate buffer pH 6.0 was prepared in doubly distilled water and was adjusted pH using 0.1 M NaOH and H_2_SO_4_.

### 2.2. Instrumentation

**Figure 1:**
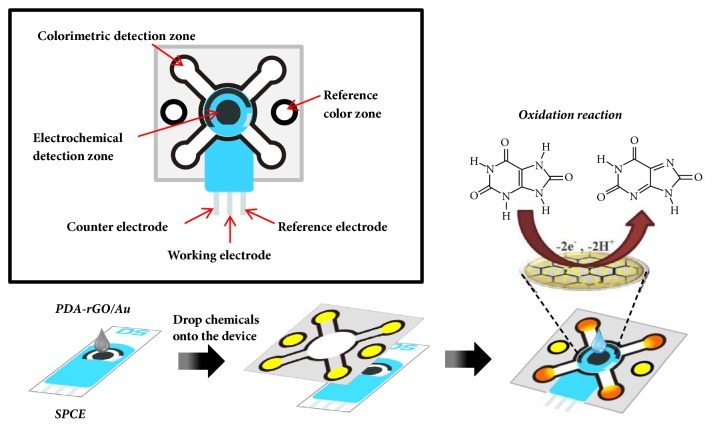
Diagram of *μ*PAD for the determination of uric acid and creatinine.

Cyclic voltammetry (CV) and square-wave voltammetry (SWV) measurements were performed using a potentiostat PGSTAT-10 (Eco Chemie, Utrecht, Netherlands) controlled by the GPES 4.9 software. SPCEs used in this study were purchased from Metrohm Autolab B.V. (DropSens). The SPCEs were composed of a three electrode composition (10 mm × 34 mm), which included a round ended carbon working electrode (4 mm in diameter), carbon counterelectrode, and silver pseudoreference electrode printed on a ceramic support. A ring shaped insulating layer around the round ended working electrode (8 mm) with a capacity of 50 *μ*L was incorporated onto the SPCEs as an electrochemical cell (Figure 1). Scanning electron microscopy (SEM) was performed using JEOL JSM-6610LV scanning electron microscope. An energy dispersive X-ray (EDX) spectrum was performed using OXFORD INCA350 that was attached with JEOL JSM-6610LV scanning electron microscope. Powder X-ray diffraction patterns were collected from 5 to 55° in 2*θ* by an X-ray diffraction (XRD) with cobalt K*α* radiation (BTX II Benchtop XRD, Olympus). UV-visible absorption spectra of creatinine were recorded by Biochrom Libra S80 Double Beam Spectrophotometer (Biochrom, UK).

### 2.3. Preparation of the PDA-rGO/Au Nanocomposite

GO was synthesized from natural graphite powder using a modified Hummer's method [20]. 500 mg of graphite powder was mixed with 60 mL of HNO_3_ and 90 mL of H_2_SO_4_. After stirring the suspension for 1 h, it was mixed in an ice bath for 1 h. Next, 250 mg of NaNO_3_ followed by 1,500 mg of KMnO_4_ was slowly added to a cooling suspension with stirring for one hour. Then, the suspension was let cooling to the room temperature prior to adding 100 mL of deionized water. The stirring was continued for overnight at room temperature. H_2_O_2_ was dropwise with stirring until gas evolution ceased and was allowed to stand overnight until GO sheets precipitation occurred. In order to remove metal ions, the GO sheets were cleaned by adding 5 M HCl into the solution and washed repeatedly with deionized water until the solution pH was neutral. The yellow-brown GO suspension was dried in an oven at 60°C. The GO suspension was characterized the physical property by TEM, SEM and XRD.

For the preparation of PDA-rGO, 100 mg of freshly prepared GO was ultrasonically dispersed in 50 mL of deionized water for 30 min to form a homogeneous GO suspension. Then, 10 mg dopamine hydrochloride dissolved in 50 mL of 10 mM Tris(hydroxymethyl)aminomethane hydrochloride solution (pH 8.5) was rapidly added to the GO suspension with stirring at 65°C for overnight. The suspension color changed from a yellow-brown color to black after overnight [21]. Then, the black solid was washed for the several times with deionized water to remove unreacted dopamine. The characterization of PDA-rGO solid physical property was obtained by TEM, SEM, and XRD.

Preparation of PDA-rGO/Au was begun by ultrasonic dispersing a 50 mg PDA-rGO slurry in 50 mL of deionized water for 20 min. Accurate quantities of 5, 10, 15, and 20 % w/w Au (HAuCl_4_·3H_2_O) were added to PDA-rGO slurry. Next, 10 mL of a 4 mg mL^−1^ NaBH_4_ solution was added dropwise with continuous stirring for 2 h. The mixture was washed thoroughly with deionized water until the solution pH was neutral. Finally, the PDA-rGO/Au suspended precipitate was collected by centrifugation and dried in an oven at 60°C. For the electrode modification, 2 mg of PDA-rGO/Au nanocomposite was dispersed in 1 mL of mixture solution (6.25% nafion solution, 20% ethanol, and 73.75% deionized water by v/v) for 30 min. After that, 5 *μ*L of the PDA-rGO/Au nanocomposite solution was dropped onto the working electrode and allowed to dry completely at room temperature for overnight (Figure 1).

### 2.4. Electrochemical Detection of Uric Acid

The cyclic voltammetric analyses were investigated by cycling the potential between -0.8 and +1.2 V at 50 mV s^−1^. The square-wave voltammetric measurements were performed by applying a potential from -1.0 to +0.8 V with pulse amplitude of 150 mV, step height of 5 mV, and square-wave frequency of 30 Hz. All experiments were operated at room temperature. The modified electrode could be used repeatedly after rinsing with distilled water.

### 2.5. Design and Fabrication of the *μ*PAD

A pattern of *μ*PADs consisted of the electrochemical detection zone, reference color zone and four colorimetric detection zones. The size of each device was approximately 2.80 × 2.80 cm^2^. For the fabrication procedure using a wax-patterning method, a pattern was printed on filter paper Whatman No.1 using wax printer (Xerox ColorQube 8870, Japan). Then, the wax-patterned paper was placed into an oven for 5 min at 105°C and cooled at room temperature. The back side of the device was covered with a transparent tape to prevent the solution from leaking out of the device. Finally, the electrochemical detection zone of device was punched as a circle hole and put onto the electrode (Figure 1).

### 2.6. Colorimetric Detection of Creatinine

The experimental process was explained in Figure 1. Firstly, 1 *μ*L of 0.04 M picric acid and 1.5 *μ*L of 5% sodium hydroxide were dropped onto each colorimetric detection and reference color zones. They were left until dry for 10 min. For analysis, 50 *μ*L of creatinine standard/sample solution was added to the electrochemical detection zone of *μ*PAD and was carried out by CV, SWV and chronoamperometry. The solution simultaneously flowed into the colorimetric detection zone by capillary force. After an incubation time for 30 min, the color change on the PADs can be observed. The color change was inspected in the colorimetric detection zone within 45 min at room temperature. The *μ*PAD image was scanned with CanoScan LiDE 700F using 600-dpi resolution, and the colors were analyzed with the ImageJ software using the whole spot. A color threshold was employed and the hue was adjusted (0-235) to remove the wax background. The images were then converted to gray scale and inverted; the blackness of color was relative to the color intensity. After the inversion, the color intensity was measured. For the blank test, the 0.1 M PBS pH 6.0 was used for a blank signal. The blank signal intensity was then subtracted from the color intensity to obtain the corrected mean intensity (∆*I*).

### 2.7. Sample Preparation

An artificial urine was prepared following Chutipongtanate* et al.* [22] by dissolving of ascorbic acid, citric acid, glucose, sodium chloride, calcium chloride, ammonium chloride, potassium chloride, urea, trisodium citrate, magnesium sulphate, sodium hydrogen carbonate, sodium sulphate, sodium hydrogen phosphate, and sodium dihydrogen phosphate in deionized water. Five levels of uric acid and creatinine were spiked in the artificial urine samples to represent the healthy (sample 1-2) and high level of the target analytes for unhealthy populations (sample 3-5). To obtain both analyte concentrations in our working linear rage, the first artificial urine sample containing 1 mM uric acid and 5 mM creatinine was made a 25-fold dilution with 0.1 M PBS pH 6.0. On the other hand, the second artificial urine sample containing 2 mM uric acid and 20 mM creatinine was made a 50-fold dilution. Likewise, the other artificial urine samples (sample 3-5) containing 4, 6, and 8 mM uric acid and 50, 100, and 200 mM creatinine were made 80-fold dilutions, respectively. Then they were analyzed by the chronoamperometry and colorimetric method. To demonstrate the application of our method, urine and creatinine in the control urine samples also were determined by our method. Liquid assayed urinalysis control samples level I (Lot. UB1519021A) and level II (Lot. UB1519022A) were obtained from Thermo Scientific (Waltham, MA). Levels of analytes such as urobilinogen, bilirubin, protein, nitrite, ketones, ascorbic acid, glucose, microalbumin, and creatinine were provided by the supplier. Prior to measurement, liquid assayed urinalysis control samples were made a 10-fold dilution with 0.1 M PBS pH 6.0.

## 3. Results and Discussion

### 3.1. Characterization of PDA-rGO/Au Nanocomposite

**Figure 2:**
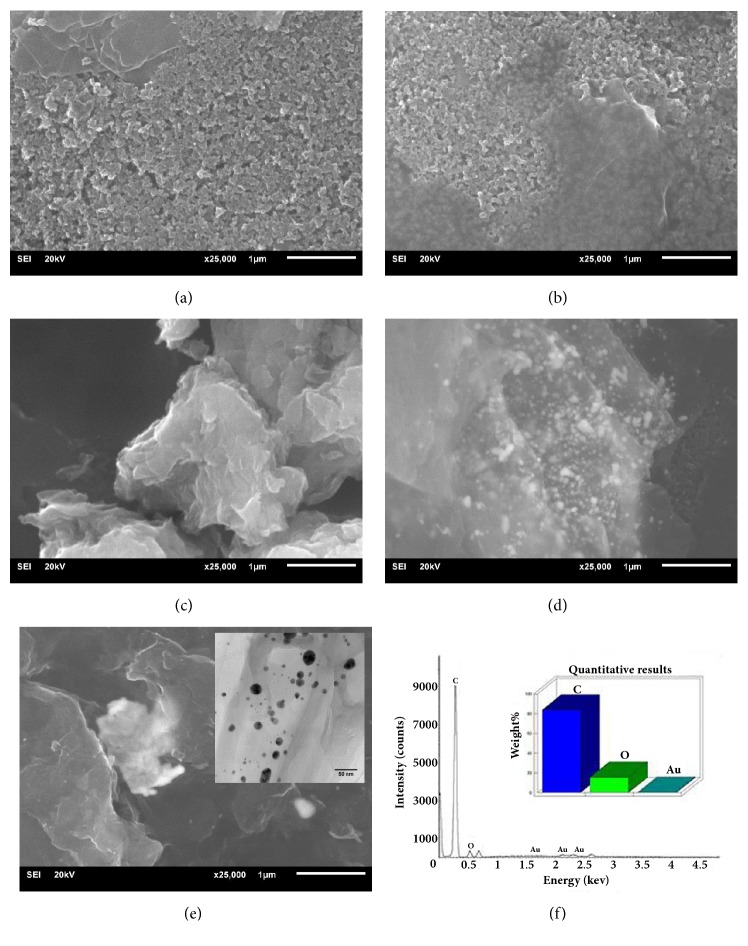
SEM images of (a) bare SPCE, (b) GO, (c) PDA-rGO, (d) rGO/Au, (e) PDA-rGO/Au (inset of Figure 2(e) shows TEM image), and (f) EDX pattern and its corresponding quantitative analysis of PDA-rGO/Au.

The morphology of the bare SPCE surface indicates the small particles dispersed throughout substrate (Figure 2(a)) that it can be referred to graphitic carbon powder as composition of the electrode. SEM images of the synthesized GO (Figure 2(b)), PDA-rGO (Figure 2(c)), rGO/Au (Figure 2(d)), and PDA-rGO/Au (Figure 2(e)) were shown. Figure 2(b) demonstrated the layers assembled with randomly as-prepared GO sheets whereas PDA-rGO composite contained no stacked rGO sheets (Figure 2(c)). The property of PDA functionalized rGO is an excellent dispersity, according to the strong hydrophilic nature of DA. Hence, PDA was covered thinly on the surface of GO sheets [23]. The surface morphology of rGO/Au composite (Figure 2(d)) was compared with the PDA-rGO/Au (Figure 2(e)). The Au aggregation in size of micrometer for the rGO/Au composite was found whereas PDA-rGO/Au was found the well dispersity of Au nanoparticles. Moreover, the deposition and distribution of Au nanoparticles on PDA-rGO/Au were investigated by TEM. Figure 2(e) inset illustrated TEM image of the nanosized particles (7.53 nm) of Au that were excellently loaded on PDA-rGO. Furthermore, EDX was performed to confirm the elemental information on the PDA-rGO/Au nanocomposite (Figure 2(f)). The spectrum confirmed the presence of exclusively carbon (C), oxygen (O), and gold (Au), which indicated the success of the composite formation with a high purity. We also confirmed the element of PDA-rGO/Au nanocomposite by XRD.  Figure S1 showed the XRD pattern of graphite, GO, PDA-rGO, and PDA-rGO/Au. XRD pattern of graphite displayed a high intensity peak around 30.9° corresponding to the graphitic structure (002) (Figure S1(a)). After oxidation, the (002) peak of graphite vanished, while a newly peak at 13.9° appeared. This peak corresponded to the (001) diffraction peak of graphene oxide (Figure S1(b)) [24]. However, the peak was not seen in PDA-rGO (Figure S1(c)) and PDA-rGO/Au nanocomposite (Figure S1(d)), indicating the GO has been reduced after the functionalization with PDA. A small peak centred at 18.8° was due to the presence of stacked graphene layers of rGO. Moreover, there were other two peaks at 44.2° and 51.5° corresponding to the (200) and (220) lattice planes of the Au. These peaks represented the characteristic of Au nanoparticles [25]. The XRD results clearly demonstrated the achievement in PDA-rGO/Au nanocomposite formation, as supposed by SEM and TEM results obtained above.

### 3.2. Effect of Scan Rate

**Figure 3:**
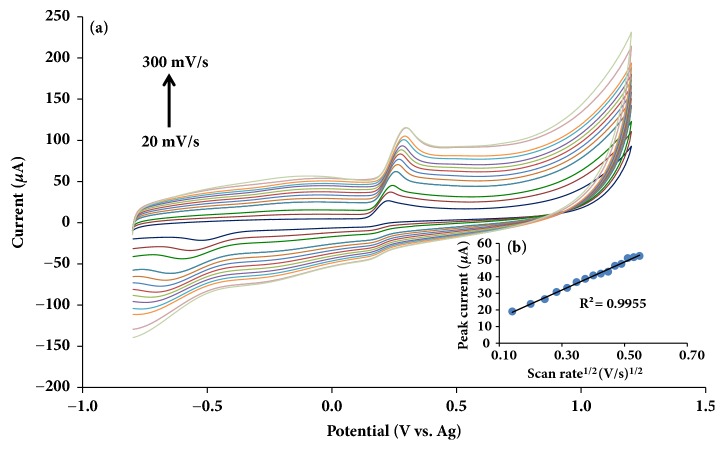
(a) Cyclic voltammograms of 1 mM uric acid in 0.1 M PBS pH 6.0 on PDA-rGO/Au with various scan rates 20, 40, 60, 80, 100, 120, 140, 160, 180, 200, 220, 240, 260, 280, and 300 mV s^−1^. (b) Plot for the anodic peak current versus the square root of the scan rate (*ν*^1/2^) in the same solution.

Effect of scan rate on the electrochemical behavior of uric acid at PDA-rGO/Au modified on SPCE was investigated by CV.  Figure 3(a) exhibited the CV responding to the PDA-rGO/Au modified on SPCE in 1 mM uric acid containing 0.1 M PBS pH 6.0 at various scan rates (20-300 mV s^−1^). It was observed that the anodic peak currents of uric acid raised and the peak potential were shifted slightly towards more positive direction with the scan rate increment. In addition, Figure 3(b) revealed a linearity between square root of the scan rates (*ν*^1/2^) and the anodic peak current of uric acid. According to Randles Sevick equation (1), the reaction involved was a mass transfer controlled [26–28]; it was stated as follows.

(1) $$\begin{array}{c} i_{p}=\left(\;2.99\times 10^{5}\;\right)n^{3/2}AD^{1/2}\nu^{1/2}C \end{array}$$

where the number of electrons exchanged during the redox process is* n*, the active area of the working electrode is* A* (cm^2^), and the diffusion coefficient and the bulk concentration of uric acid are* D* (cm^2^  s^−1^) and* C* (mol cm^−3^), respectively. The voltage scan rate (V s^−1^) is *ν*.

The result confirmed that the oxidation behavior of uric acid at PDA-rGO/Au modified electrode is controlled by a diffusion controlled electrochemical process [29].

### 3.3. Effect of %Au Loading on PDA-rGO

**Figure 4:**
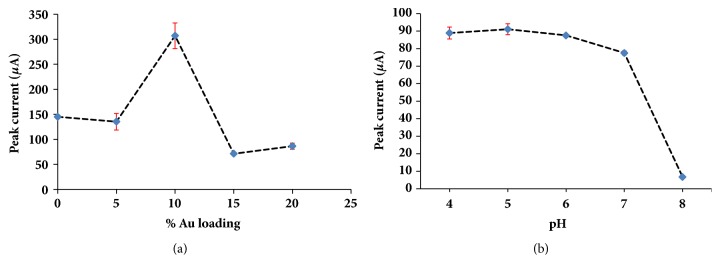
(a) Effect of gold loading on supported material on the anodic peak currents of 1 mM uric acid detection. (b) Effect of pH on the anodic peak currents of 1 mM uric acid detection.

The %Au loading on PDA-rGO has an effect on the oxidation of uric acid due to its agglomeration at the high %Au or the low conductivity at the low %Au. Thus, the %Au loading at 0, 5, 10, 15, and 20% w/w were studied.  Figure S2 displayed the SWVs of 1 mM uric acid of the various % loading of Au on PDA-rGO. The oxidation current increased as the %Au loading increased until it reached at 10% w/w (Figure 4(a)) because of the excess amount of Au nanoparticles on PDA-rGO resulting in the agglomeration of Au nanoparticles. Thus, the 10% w/w loading of Au on PDA-rGO was chosen for the next study.

### 3.4. Effect of pH

In the practical samples, pH of urine was varied from 4.6 to 8.0 so the effect of pH on the uric acid determination was studied. The effect of pH on the anodic peak current of 1 mM uric acid at PDA-rGO/Au modified electrode was monitored by SWV. The result was shown in Figure S3.  Figure 4(b) showed the relationship between anodic peak current as a function of pH. The anodic peak current appeared not to be significantly different from pH 4.0 to 6.0 and then decreased from 7.0 to 8.0. The deprotonation of uric acid occurred when the pH was above 6.0 (pK_a_ = 5.6) and could repulse negatively charged composite modified electrode. This causes a low anodic current of uric acid at pH above 6. Effects of pH on the anodic peak current at pH 4, 5, and 6 were compared by one-way ANOVA method as shown in Table S1. The anodic peak current for the uric acid determination was no significant (p > 0.05) differences between pH 4, 5, and 6. The pH 6 is similar to real physiological conditions (pH 5.5-6.5). Therefore, pH 6.0 was selected as an optimum pH for the next experiments.

### 3.5. Electrochemical Behavior of Uric Acid on PDA-rGO/Au Nanocomposite Modified Electrode

**Figure 5:**
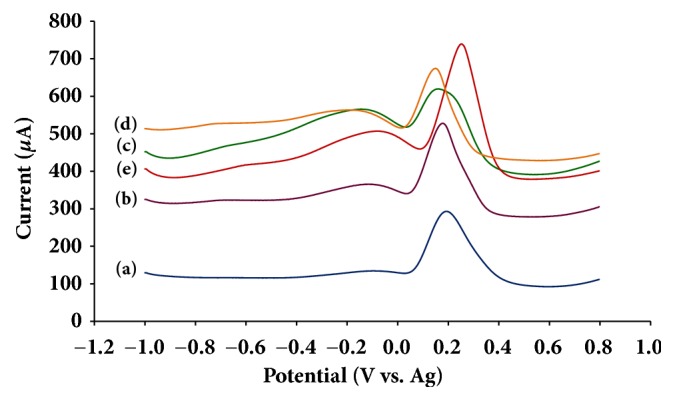
Square-wave voltammograms of (a) bare SPCE, (b) GO/SPCE, (c) PDA-rGO/SPCE, (d) GO/Au/SPCE, and (e) PDA-rGO/Au/SPCE in 0.1 M PBS pH 6.0 containing 1 mM uric acid.

The electrochemical oxidations of uric acid on different modified electrodes have been studied. The SWV profiles of 1 mM uric acid on modified electrode were presented in Figure 5. It can be observed that the SWV of 1 mM uric acid on GO modified SPCE showed an enhanced anodic current more than the bare SPCE (Figures 5(a)-5(b)). It increased the conductivity and provided a higher surface area. The GO/Au modified SPCE also generated a lower oxidation potential of uric acid (around +0.20 V versus Ag/AgCl ink) compared to the bare GO electrode (Figure 5(d)). It indicated that the Au decorated on GO could undergo the catalytic oxidation of uric acid at the GO electrode surface. PDA gave a suitable environment for the adsorption of Au onto PDA-rGO without Au aggregation, whereas the GO/Au modified SPCE without PDA functionalization displayed a strong aggregation of Au. Moreover, uric acid could easily interact with PDA by intermediate hydrogen bonds between -OH group and pyrrolic -NH of PDA. Therefore, PDA-rGO/Au modified SPCE showed a further increased anodic current than those electrodes (Figure 5(e)).

### 3.6. Interference of PDA-rGO/Au Nanocomposite

The interference effects of the PDA-rGO/Au modified SPCE were studied using other possible interference species in the urine system. Under the optimized conditions, 1 mM uric acid mixed with the common species in urine consisting of 1 mM ascorbic acid, 5 mM citric acid, 10 mM calcium chloride, 1 mM creatinine, 5 *μ*M dopamine, 1 mM glucose, 500 mM potassium chloride, 500 mM sodium chloride, 1 mM ammonium chloride, and 100 mM urea were evaluated. The detailed results were provided in Figure S4. The anodic current of uric acid mixed with those species fell in the range of 94-114% when compared to uric acid without mixing those species. This indicated that the obtained PDA-rGO/Au as a uric acid sensor exhibited high selectivity; thus it can be a great promise in practical application.

### 3.7. Analytical Performance of PDA-rGO/Au Nanocomposite Modified Electrode

**Figure 6:**
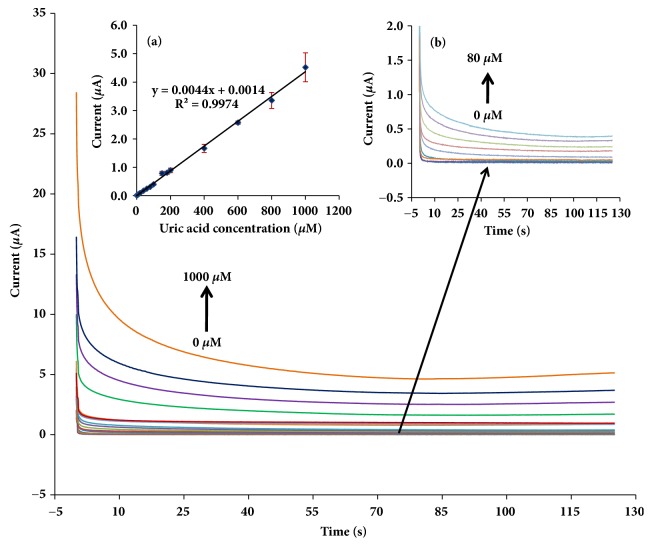
Chronoamperograms at the PDA-rGO/Au in 0.1 M PBS pH 6.0 containing different concentrations of uric acid at +0.25 V versus Ag/AgCl ink. The inset shows (a) the calibration curve for uric acid detection, n = 3, and (b) the enlarged chronoamperograms of uric acid from 0 to 80 *μ*M.

Chronoamperometry (CA) was used instead of SWV because it is an easier detection mode to implement for point-of-care testing and has higher sensitivity.  Figure 6 illustrated chronoamperograms with the increasing uric acid concentration in the range of 2.5-1,000 *μ*M, at the potential level of +0.25 V versus Ag/AgCl ink. The linear plot of anodic currents was recorded at 100 s as a steady state. The coefficients of linearity (R^2^) were found at 0.9974. The detection limit was found to be 0.74 *μ*M by calculated concentration from the signal at three times of the standard deviation for a blank (n = 10). Our analytical performance is comparable to others in Table 1. The linear response range obtained from our sensor is wider than the previous free-enzymatic sensors except TCPP-rGO sensor [30] but our detection limit was lower than the TCPP-rGO sensor. PDA could be used to avoid Au and GO aggregation which leads to increasing of the electrochemical activity of GO on the electrochemical sensing of uric acid. When the analytical performance of our sensor was compared with the Au/rGO modified on glassy carbon electrode, our sensors are better in the both term of the wider linearity and lower detection limit. Furthermore, the SPCE exhibited over advantages than other bulk electrodes, such as disposability, low-cost, simplicity, enabling the use of microvolumes of samples without complementary glassware cell, and the possibility of coupling with *μ*PADs. We also compromised both wide linear range and low detection limit so our sensor is useful for clinical diagnostic where the normal level of uric acid is 120 to 420 *μ*M for serum and 1.48 to 4.43 mM for urine.

Additionally, the repeatability of PDA-rGO/Au modified electrode was demonstrated at 600 *μ*M uric acid (n=10) at same electrode. We obtained the relative standard deviations (RSD) of 3.25% at this concentration detected at the same electrode. The reproducibility of the proposed electrode was investigated by means of the data obtained from five separately prepared PDA-rGO/Au modified SPCE in 600 *μ*M of uric acid. The relative standard deviation (RSD) of this concentration detected by five separately prepared PDA-rGO/Au modified SPCE was found to be 3.31% (n=5). The results indicated that repeatability and reproducibility of the proposed electrode were in an acceptable level with AOAC International (less than 3.70%) [31]. In addition, the storage stability of the PDA-rGO/Au modified electrode was studied during 7 storage days.  Figure S5 showed the stability for a week towards the uric acid detection. The anodic current remained fairly stable within the first week of the electrode fabrication. After 7 days, only about 10% decrease was observed in the anodic current of the proposed electrode. These results supported that this modified electrode reveals good repeatability, reproducibility and stability in the electrooxidation of uric acid.

### 3.8. Colorimetric Measurement and UV-Visible Spectroscopy of Creatinine

The Jaffé reaction under alkaline condition was applied to detect creatinine levels. Creatine reacts with picric acid to form a Janovsky complex (orange color) in an alkaline medium, which can enhance the specificity for creatinine detection (equation (2)).

(2) Creatinine+Picric  acidyellow⟶alkalineJanovsky  complexorange

The UV-visible absorption spectra of picric acid in a sodium hydroxide solution presented at 355 and 410 nm of absorption bands (Figure S6(b)). After the addition of 1 and 2 mM creatinine to the picric acid solution, the two absorption bands were clearly increased, which indicated the Janovsky complex from reaction of creatinine with picric acid (Figure S6(c-d)). These results were in good accepting with the visualized measurement. 

### 3.9. Specificity of Creatinine Determination

The specificity of the colorimetric measurement of creatinine was evaluated using the various substance commonly found in urine. The common species in urine consisting of 1 mM uric acid, 1 mM ascorbic acid, 5 mM citric acid, 10 mM calcium chloride, 0.005 mM dopamine, 1 mM glucose, 500 mM potassium chloride, 500 mM sodium chloride, 1 mM ammonium chloride, and 100 mM urea were tested. The results are presented in Figure S7. Only 4 mM creatinine can change color from yellow to orange. Hence, the proposed method was obviously specificity for the determination of creatinine in urine samples.

### 3.10. Analytical Performance of Creatinine

**Figure 7:**
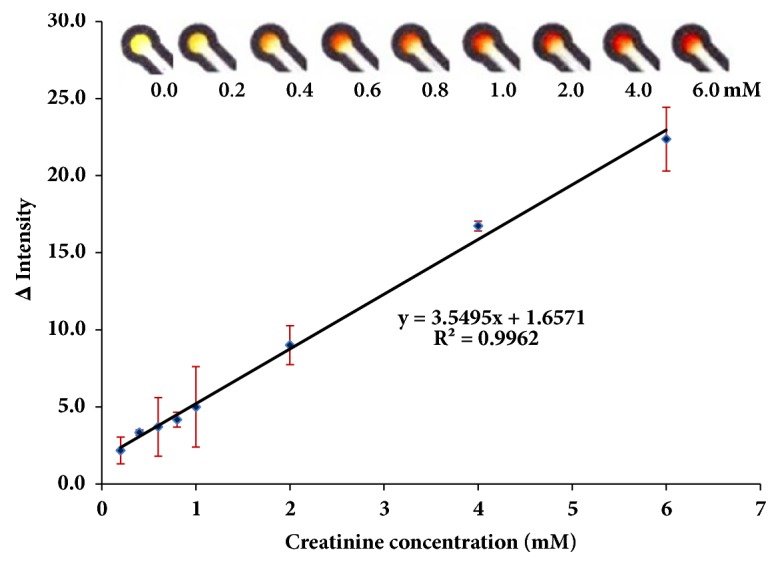
Calibration plots for the creatinine determination (n=3).

A calibration curve for the determination of creatinine was achieved by colorimetric *μ*PAD. A linear correlation between ∆Intensity (creatinine signal minus blank signal) and creatinine concentration was observed in the range of 0.20-6.0 mM (r^2^ = 0.9962) which the color changed from yellow color to orange color (Figure 7). The detection limit for creatinine was calculated by three times of the standard deviation of 0.20 mM creatinine divided by the slope of the linear range. The detection limit of creatinine was found to be 180 *μ*M (n=12).

### 3.11. Application in Real Samples

In order to estimate the practicable performance of this method, the electrochemical detection using PDA-rGO/Au modified with SPCE and the colorimetric detection based on Jaffé reaction were utilized for the quantitative measurement of uric acid and creatinine in the control urine samples. The analytical results were reported in Table 2. The recovery percentages of uric acid and creatinine in the control urine samples were found in the range of 98-102% and 99-104%, respectively. %RSD of these experiments for the uric acid and creatinine determination was established in the range of 2.57-3.58% and 2.31-3.41%, respectively. Moreover, the artificial urine samples were investigated and the results are shown in Table S2. Furthermore, the ratios of uric acid to creatinine were examined in the control urine samples and the recovery percentages were found in the range of 98-101% (Table S3). It was proved that the proposed method was dependable. Thus, our designed method is applicable for the determination of uric acid and creatinine in urine samples.

## 4. Conclusions

The portable and disposable nonenzymatic sensor for uric acid and creatinine has been firstly developed. The PDA-rGO/Au nanocomposite modified on SPCEs has been successfully performed in the nonenzymatic sensor for uric acid. The specific determination of creatinine has been developed using colorimetric detection by Jaffé reaction. Under the optimal conditions, the detection limit of uric acid was 0.74 *μ*M with wide linear range of 2.5 to 1,000 *μ*M achieved by electrochemical detection, whereas the detection limit of creatinine was 180 *μ*M with a linear dynamic range from 0.20 to 6.0 mM achieved by colorimetric detection. These proposed methods are rapid, enzyme-free sensor, cost effective, single drop sample for the simultaneous determination of creatinine and uric acid, and especially easy to use without any complicated instruments. Thus, they could be beneficial for future clinical laboratory analysis.

## Figures and Tables

**Table 1:** Comparative analytical performance of this as-prepared sensor and some others for the uric acid determination.

Electrode	Modified	Method	Linear range (*μ*M)	LOD (*μ*M)	Ref.
GCE	Β-CD/SPNAANI film	DPV	10-350	2.70	[32]
GCE	MWCNT-PEDOT film	DPV	10-250	10.0	[33]
GCE	Au/rGO	DPV	8.8-53	1.80	[34]
Sonogel-CE	Cysteine	SWV	10-100	10.0	[35]
GCE	AuNPs-GO	DPV	20-260	20.0	[36]
GCE	TCPP-rGO	Amperometry	20-5000	1.00	[30]
GCE	rGO-ZnO	DPV	3-330	1.08	[37]
SPCE	PDA-rGO/Au	Chronoamperometry	2.5-1000	0.74	This works

**Table 2:** Determination of uric acid and creatinine in the control urine samples.

Analyte	Liquid Assayed Urinalysis Control level I (Normal)					Liquid Assayed Urinalysis Control level II (Abnormal)				
	Certified (mM)	Added (mM)	Found (mM)	% Recovery	% RSD (n=3)	Certified (mM)	Added (mM)	Found (mM)	% Recovery	% RSD (n=3)
Uric acid	-	4.00	3.92	98	2.57	-	6.00	6.13	102	3.58
Creatinine	8.80	-	8.72	99	2.31	26.50	-	27.47	104	3.41

## Data Availability

The data used to support the findings of this study are available from the corresponding author upon request.

## Supplementary Materials

Supplementary Materials As supplementary materials Figures S1-S7 and Table S1-S3 are included. Figure S1: XRD patterns of graphite, GO, PDA-rGO, and PDA-rGO/Au. Figure S2: square-wave voltammograms of 1 mM uric acid at bare SPCE and various %loading of Au on PDA-rGO. Figure S3: square-wave voltammograms of 1 mM uric acid in the deferential pH. Figure S4: histogram for interferents of uric acid at the PDA-rGO/Au in 0.1 M PBS pH 6.0. Figure S5: electrode stability in response to 1 mM uric acid for 7 days. Figure S6: UV-visible spectra of the picric acid in NaOH solution with/without of creatinine. Figure S7: histogram for interferents of creatinine in 0.1 M PBS pH 6.0. Table S1: one-way ANOVA by SPSS program for pH effect on the uric acid detection. Table S2: determination of uric acid and creatinine in the artificial urine samples. Table S3: determination of uric acid to creatinine ratio in the control urine samples.

## References

[1] Nishida Y. (1992). Relation between creatinine and uric acid excretion. *Annals of the Rheumatic Diseases*.

[2] Wang C., Yuan R., Chai Y., Zhang Y., Hu F., Zhang M. (2011). Au-nanoclusters incorporated 3-amino-5-mercapto-1,2,4-triazole film modified electrode for the simultaneous determination of ascorbic acid, dopamine, uric acid and nitrite. *Biosensors and Bioelectronics*.

[3] Johnson R. J., Kang D.-H., Feig D. (2003). Is there a pathogenetic role for uric acid in hypertension and cardiovascular and renal disease?. *Hypertension*.

[4] Abellán-Llobregat A., Vidal L., Rodríguez-Amaro R., Berenguer-Murcia Á., Canals A., Morallón E. (2017). Au-IDA microelectrodes modified with Au-doped graphene oxide for the simultaneous determination of uric acid and ascorbic acid in urine samples. *Electrochimica Acta*.

[5] Talalak K., Noiphung J., Songjaroen T., Chailapakul O., Laiwattanapaisal W. (2015). A facile low-cost enzymatic paper-based assay for the determination of urine creatinine. *Talanta*.

[6] Songjaroen T., Maturos T., Sappat A., Tuantranont A., Laiwattanapaisal W. (2009). Portable microfluidic system for determination of urinary creatinine. *Analytica Chimica Acta*.

[7] Hanif S., John P., Gao W., Saqib M., Qi L., Xu G. (2016). Chemiluminescence of creatinine/H_2_O_2_/Co^2+^ and its application for selective creatinine detection. *Biosensors and Bioelectronics*.

[8] Mazzali M., Hughes J., Kim Y.-G. (2001). Elevated uric acid increases blood pressure in the rat by a novel crystal-independent mechanism. *Hypertension*.

[9] Kutzing M. K., Firestein B. L. (2008). Altered uric acid levels and disease states. *The Journal of Pharmacology and Experimental Therapeutics*.

[10] George S. K., Dipu M. T., Mehra U. R., Singh P., Verma A. K., Ramgaokar J. S. (2006). Improved HPLC method for the simultaneous determination of allantoin, uric acid and creatinine in cattle urine. *Journal of Chromatography B*.

[11] Muñoz J. A., López-Mesas M., Valiente M. (2010). Development and validation of a simple determination of urine metabolites (oxalate, citrate, uric acid and creatinine) by capillary zone electrophoresis. *Talanta*.

[12] Kwon W., Kim J. Y., Suh S., In M. K. (2012). Simultaneous determination of creatinine and uric acid in urine by liquid chromatography-tandem mass spectrometry with polarity switching electrospray ionization. *Forensic Science International*.

[13] Miyake M., Shibukawa A., Nakagawa T. (1991). Simultaneous determination of creatinine and uric acid in human plasma and urine by micellar electrokinetic chromatography. *Journal of Separation Science*.

[14] Fang B., Feng Y., Wang G., Zhang C., Gu A., Liu M. (2011). A uric acid sensor based on electrodeposition of nickel hexacyanoferrate nanoparticles on an electrode modified with multi-walled carbon nanotubes. *Microchimica Acta*.

[15] Sun C.-L., Chang C.-T., Lee H.-H. (2011). Microwave-assisted synthesis of a core-shell MWCNT/GONR heterostructure for the electrochemical detection of ascorbic acid, dopamine, and uric acid. *ACS Nano*.

[16] Fritea L., Tertiş M., Cristea C., Cosnier S., Săndulescu R. (2015). Simultaneous Determination of Ascorbic and Uric Acids in Urine Using an Innovative Electrochemical Sensor Based on *β*-Cyclodextrin. *Analytical Letters*.

[17] Rajamani A. R., Kannan R., Krishnan S. (2015). Electrochemical sensing of dopamine, uric acid and ascorbic acid using tRGO-TiO_2_ nanocomposites. *Journal of Nanoscience and Nanotechnology*.

[18] Fu L., Lai G., Chen G., Lin C., Yu A. (2016). Microwave Irradiation-assisted exfoliation of boron nitride nanosheets: a platform for loading high density of nanoparticles. *ChemistrySelect*.

[19] Du J., Yue R., Yao Z. (2013). Nonenzymatic uric acid electrochemical sensor based on graphene-modified carbon fiber electrode. *Colloids and Surfaces A: Physicochemical and Engineering Aspects*.

[20] Hummers W. S., Offeman R. E. (1958). Preparation of graphitic oxide. *Journal of the American Chemical Society*.

[21] Ren F., Zhai C., Zhu M. (2015). Facile synthesis of PtAu nanoparticles supported on polydopamine reduced and modified graphene oxide as a highly active catalyst for methanol oxidation. *Electrochimica Acta*.

[22] Chutipongtanate S., Thongboonkerd V. (2010). Systematic comparisons of artificial urine formulas for in vitro cellular study. *Analytical Biochemistry*.

[23] Palanisamy S., Thangavelu K., Chen S.-M., Thirumalraj B., Liu X.-H. (2016). Preparation and characterization of gold nanoparticles decorated on graphene oxide@polydopamine composite: Application for sensitive and low potential detection of catechol. *Sensors and Actuators B: Chemical*.

[24] Mishra A. K., Ramaprabhu S. (2011). Carbon dioxide adsorption in grephene sheets. *AIP Advances*.

[25] Yanqiu J., Xiuxiu Y., Qiu Y. (2016). Determination of nicotine in tobacco products based on mussel-inspired reduced graphene oxide-supported gold nanoparticles. *Scientific Reports*.

[26] Bard A. J., Faulkner L. R. (2001). *Fundamentals and Applications: Electrochemical Methods*.

[27] Laoire C. O., Mukerjee S., Abraham K. M., Plichta E. J., Hendrickson M. A. (2009). Elucidating the mechanism of oxygen reduction for lithium-air battery applications. *The Journal of Physical Chemistry C*.

[28] Keithley R. B., Takmakov P., Bucher E. S. (2011). Higher sensitivity dopamine measurements with faster-scan cyclic voltammetry. *Analytical Chemistry*.

[29] Cao X., Cai X., Feng Q., Jia S., Wang N. (2012). Ultrathin CdSe nanosheets: Synthesis and application in simultaneous determination of catechol and hydroquinone. *Analytica Chimica Acta*.

[30] Yang Y., Sun R., Li M., Geng B., Deng J., Tang M. (2016). Porphyrin functionalized graphene for sensitive electrochemical detection of uric acid. *International Journal of Electrochemical Science*.

[31] Latimer Jr G. W. (2016). Guidelines for standard method performance requirements: official methods of analysis. *AOAC International*.

[32] Wu S., Wang T., Gao Z., Xu H., Zhou B., Wang C. (2008). Selective detection of uric acid in the presence of ascorbic acid at physiological pH by using a *β*-cyclodextrin modified copolymer of sulfanilic acid and *N*-acetylaniline. *Biosensors and Bioelectronics*.

[33] Lin K.-C., Tsai T.-H., Chen S.-M. (2010). Performing enzyme-free H2O2 biosensor and simultaneous determination for AA, DA, and UA by MWCNT-PEDOT film. *Biosensors and Bioelectronics*.

[34] Wang C., Du J., Wang H. (2014). A facile electrochemical sensor based on reduced graphene oxide and Au nanoplates modified glassy carbon electrode for simultaneous detection of ascorbic acid, dopamine and uric acid. *Sensors and Actuators B: Chemical*.

[35] Choukairi M., Bouchta D., Bounab L. (2015). Electrochemical detection of uric acid and ascorbic acid: Pplication in serum. *Journal of Electroanalytical Chemistry*.

[36] Imran H., Manikandan P. N., Dharuman V. (2015). Facile and green synthesis of graphene oxide by electrical exfoliation of pencil graphite and gold nanoparticle for non-enzymatic simultaneous sensing of ascorbic acid, dopamine and uric acid. *RSC Advances*.

[37] Zhang X., Zhang Y.-C., Ma L.-X. (2016). One-pot facile fabrication of graphene-zinc oxide composite and its enhanced sensitivity for simultaneous electrochemical detection of ascorbic acid, dopamine and uric acid. *Sensors and Actuators B: Chemical*.

