# Supplementary material for: Disposable Nonenzymatic Uric Acid and Creatinine Sensors Using μPAD Coupled with Screen-Printed Reduced Graphene Oxide-Gold Nanocomposites
Source: Int J Anal Chem. 2019 Feb 3;2019:3457247. doi: 10.1155/2019/3457247 (PMC6377961; doi:10.1155/2019/3457247)
Supplement: Supplementary Materials — As supplementary materials Figures S1-S7 and Table S1-S3 are included. Figure S1: XRD patterns of graphite, GO, PDA-rGO, and PDA-rGO/Au. Figure S2: square-wave voltammograms of 1 mM uric acid at bare SPCE and various %loading of Au on PDA-rGO. Figure S3: square-wave voltammograms of 1 mM uric acid in the deferential pH. Figure S4: histogram for interferents of uric acid at the PDA-rGO/Au in 0.1 M PBS pH 6.0. Figure S5: electrode stability in response to 1 mM uric acid for 7 days. Figure S6: UV-visible spectra of the picric acid in NaOH solution with/without of creatinine. Figure S7: histogram for interferents of creatinine in 0.1 M PBS pH 6.0. Table S1: one-way ANOVA by SPSS program for pH effect on the uric acid detection. Table S2: determination of uric acid and creatinine in the artificial urine samples. Table S3: determination of uric acid to creatinine ratio in the control urine samples. [file 3457247.f1.docx]

International Journal of Analytical Chemistry

**Supplementary Materials**

# Disposable Non-enzymatic Uric Acid and Creatinine Sensors Using µPAD Coupled with Screen-Printed Reduced Graphene Oxide-Gold Nanocomposites

Kamolwich Income,^1,2^ Nalin Ratnarathorn,^1^ Napassawan Khamchaiyo,^1^ Chanut Srisuvo,^1^ Leela Ruckthong,^1^ and Wijitar Dungchai^1^

^1^ Organic Synthesis, Electrochemistry & Natural Product Research Unit, Department of Chemistry, Faculty of Science, King Mongkut’s University of Technology Thonburi, 126 Pracha-utid Road, Bang Mod, Thungkru, Bangkok, 10140, Thailand.

^2^ Department of Primary Industries and Mines, Ministry of Industry, Bangkok 10400, Thailand.

Correspondence should be addressed to Wijitar Dungchai; wijitar.dun@kmutt.ac.th


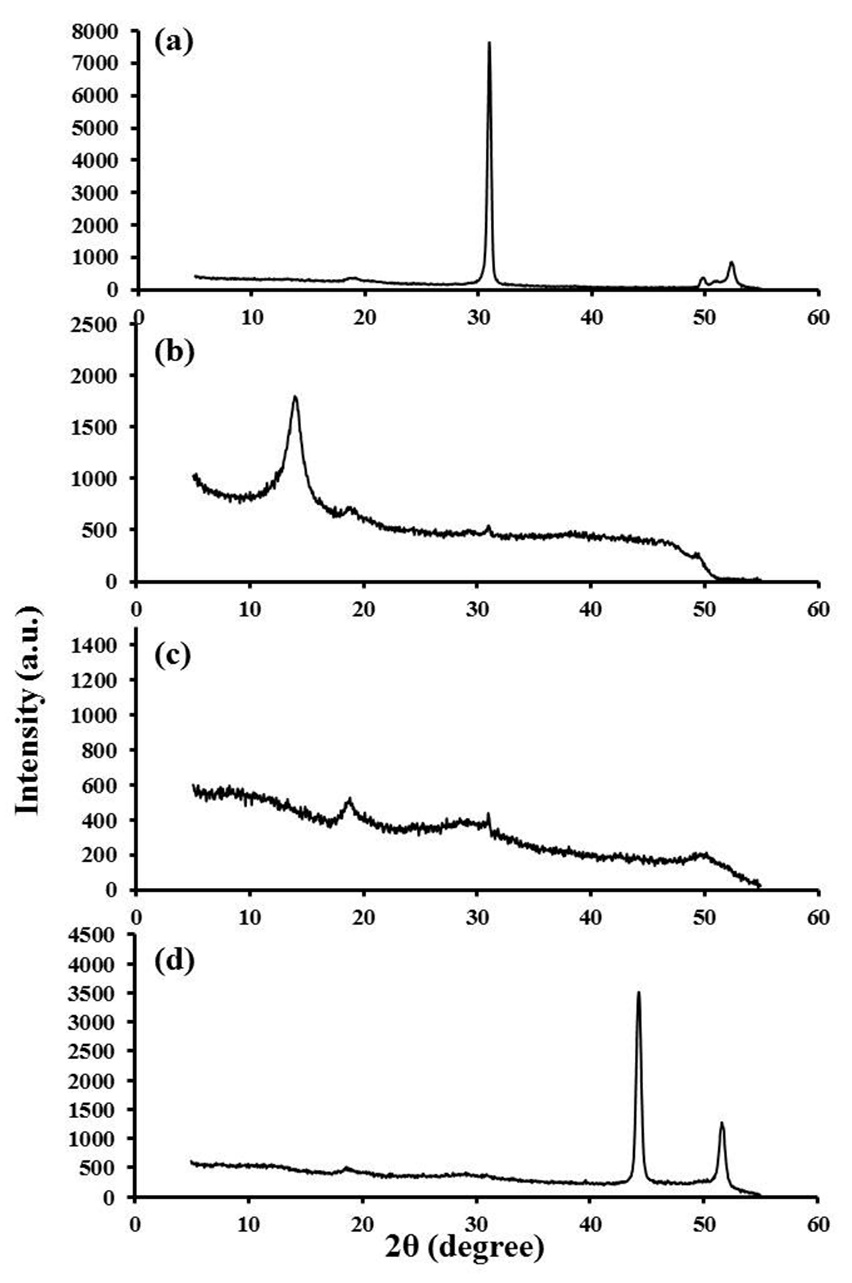


Figure S1: XRD patterns of (a) graphite, (b) GO, (c) PDA-rGO and (d) PDA-rGO/Au.


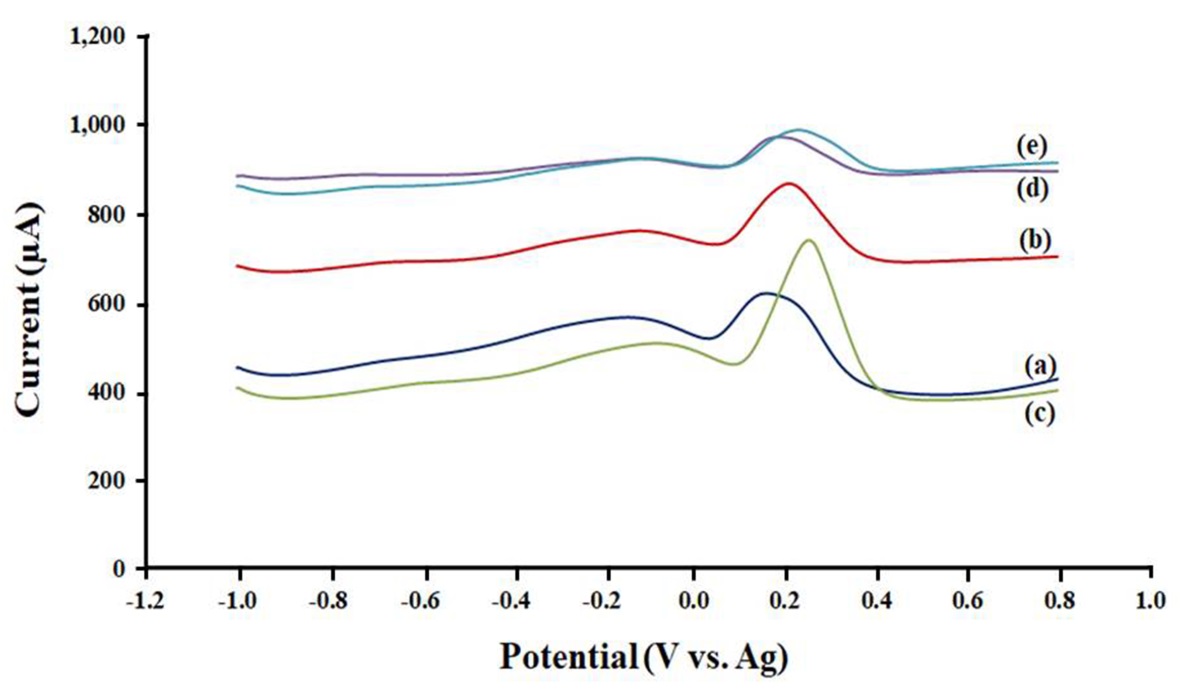


Figure S2: Square-wave voltammograms of 1 mM uric acid in 0.1 M PBS pH 6.0 at (a) bare SPCE, and (b) 5% Au, (c) 10% Au, (d) 15% Au (e) 20% Au loading on PDA-rGO.


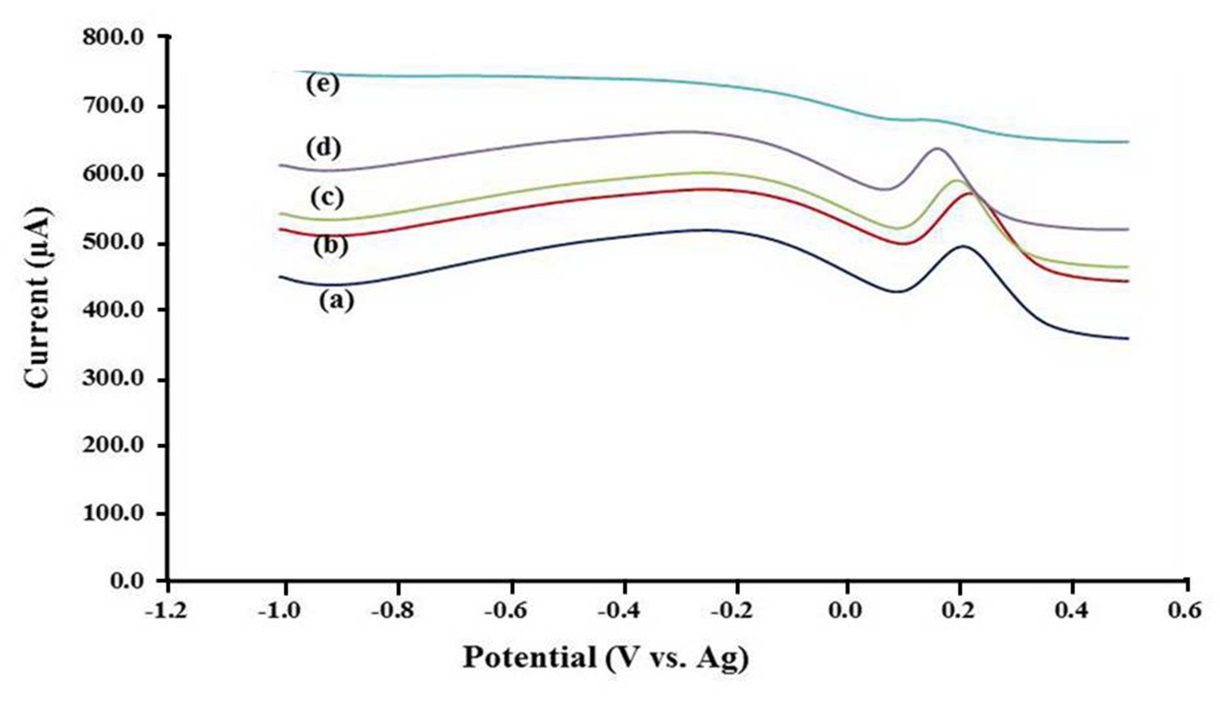


Figure S3: Square-wave voltammograms of 1 mM uric acid in the deferential pH at (a) 4.0, (b) 5.0, (c) 6.0, (d) 7.0 and (e) 8.0.

**
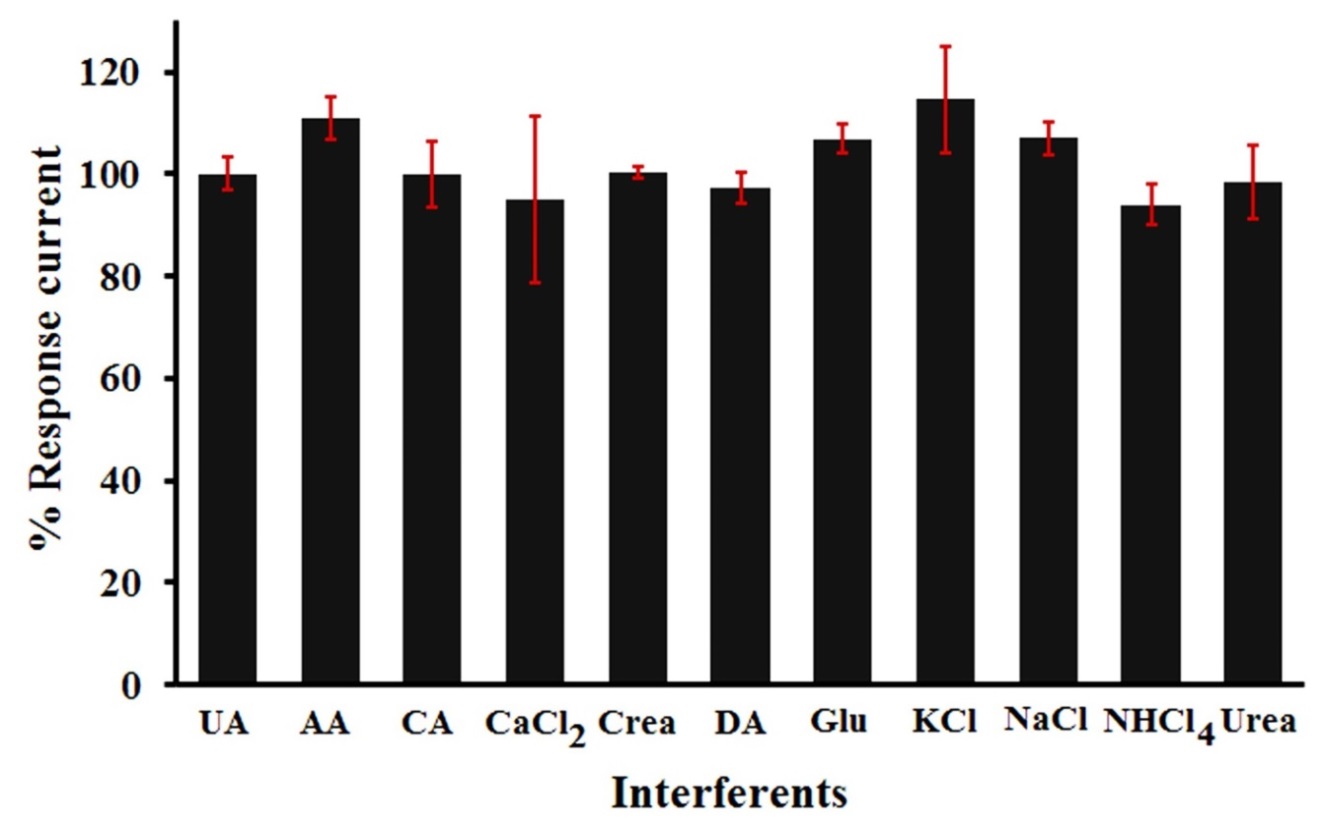
**

Figure S4: Histogram for interferents (1 mM ascorbic acid (AA), 5 mM citric acid (CA), 10 mM calcium chloride (CaCl_2_), 1 mM creatinine (Crea), 0.005 mM dopamine (DA), 1 mM glucose (Glu), 500 mM potassium chloride (KCl), 500 mM sodium chloride (NaCl), 1 mM ammonium chloride (NH_4_Cl) and 100 mM Urea) of 1 mM uric acid at the PDA-rGO/Au in 0.1 M PBS pH 6.0.


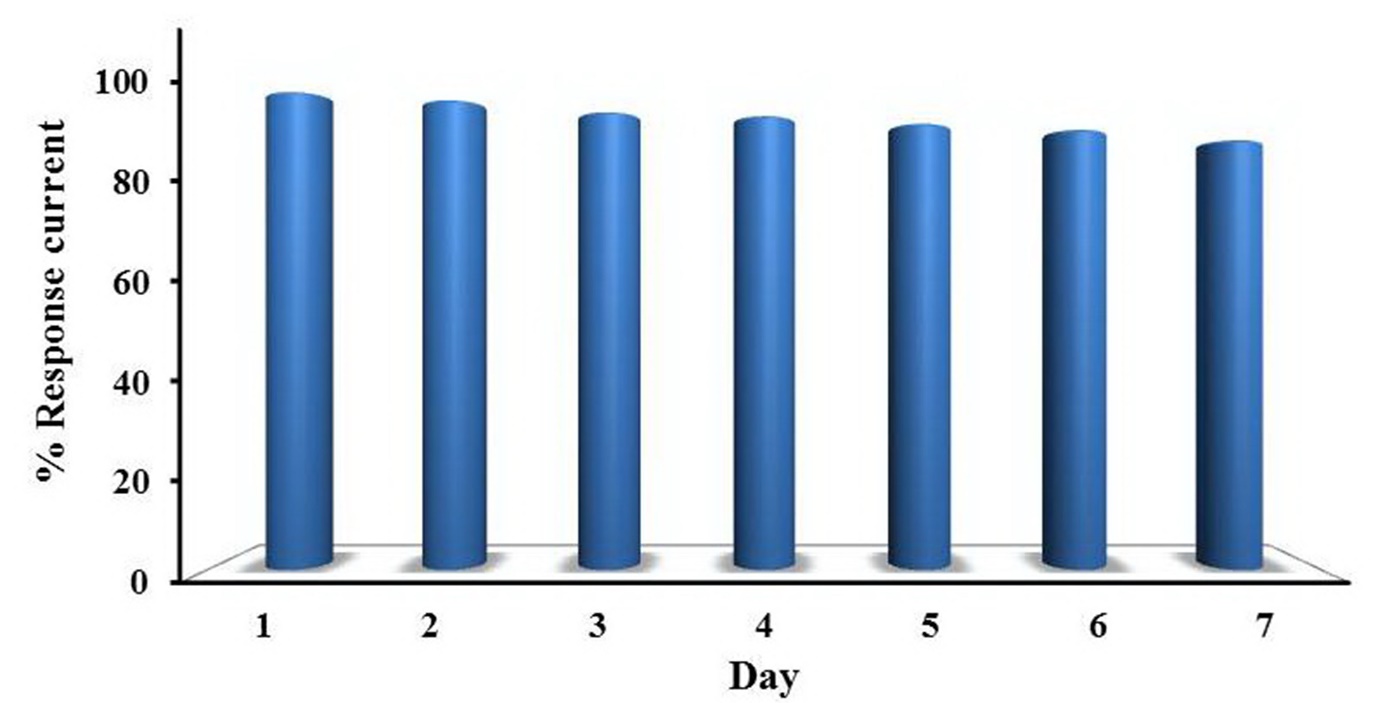


Figure S5: Electrode stability in response to 1 mM uric acid for 7 days.


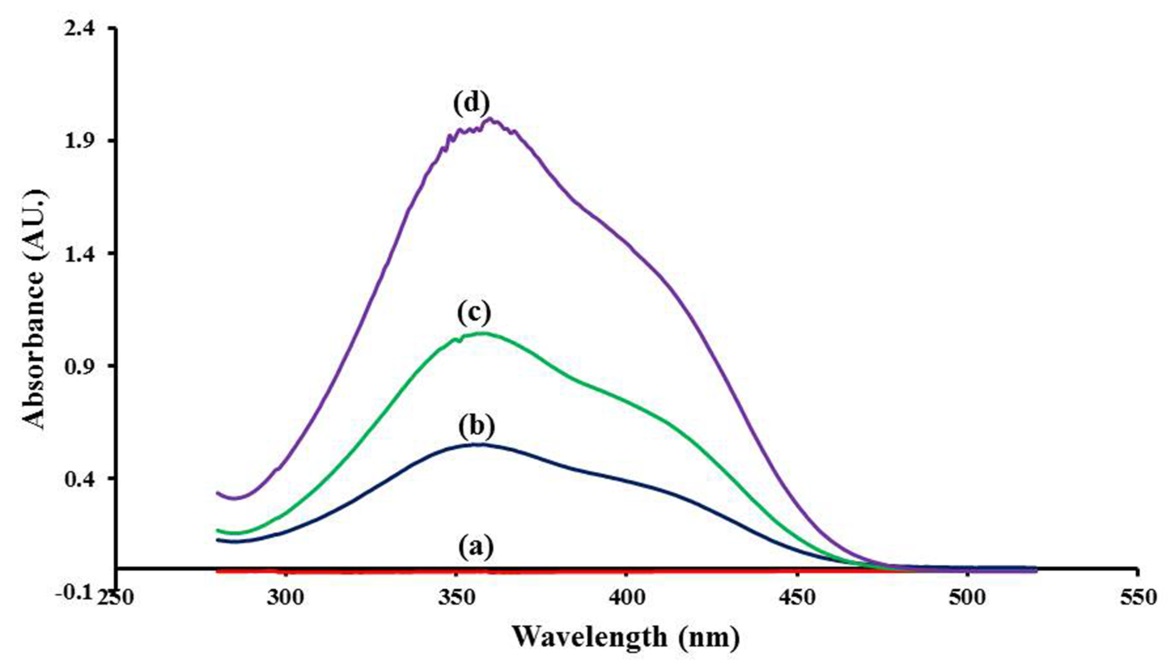


Figure S6: UV-visible spectra of (a) 1 mM creatinine, (b) 0.04 M picric acid in 5% NaOH, (c) 0.04 M picric acid in 5% NaOH with 1 mM creatinine and (d) 0.04 M picric acid in 5% NaOH with 2 mM creatinine.


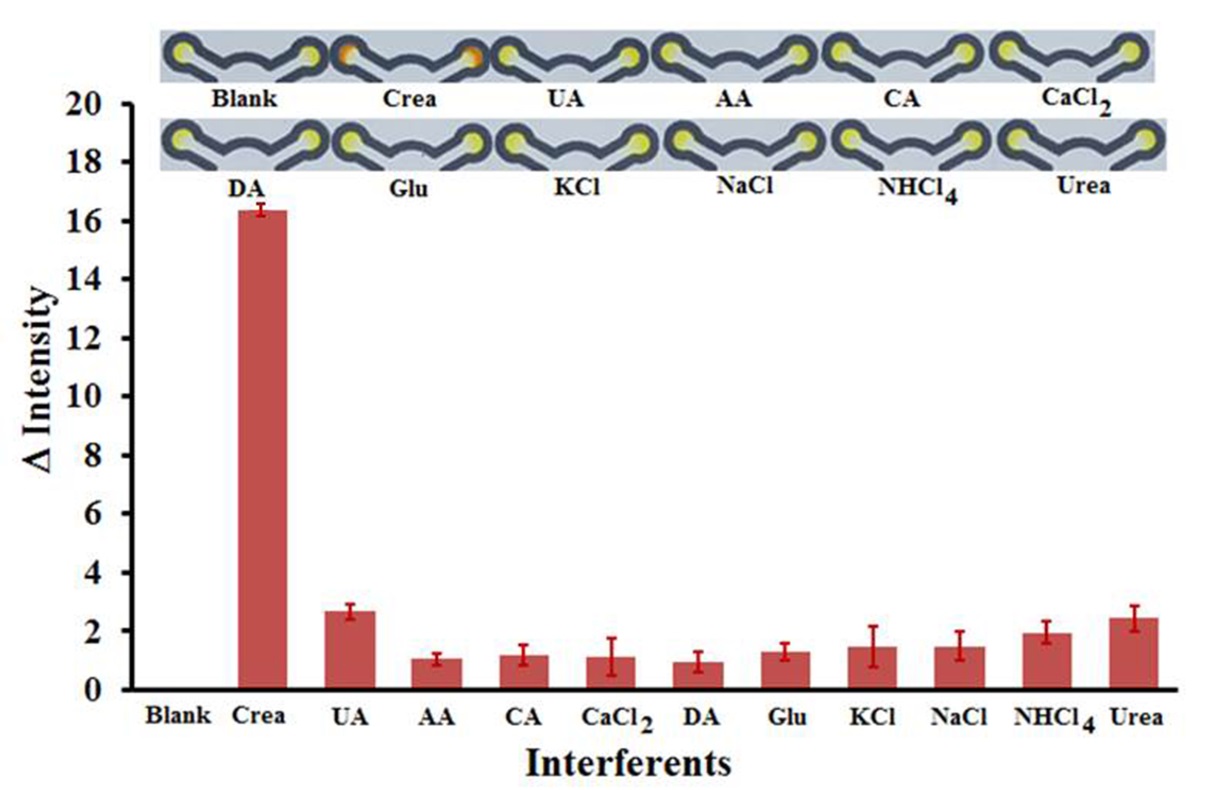


Figure S7: Histogram for interferents (1 mM uric acid (UA), 1 mM ascorbic acid (AA), 5 mM citric acid (CA), 10 mM calcium chloride (CaCl_2_), 0.005 mM dopamine (DA), 1 mM glucose (Glu), 500 mM potassium chloride (KCl), 500 mM sodium chloride (NaCl), 1 mM ammonium chloride (NH_4_Cl) and 100 mM Urea) of 4 mM creatinine in 0.1 M PBS pH 6.0.

Table S1: One-way ANOVA by SPSS program for pH effect on the uric acid detection.

**Descriptive**

| pH | N | Mean | Std. Deviation | Std. Error | 95% Confidence Interval for Mean | | Minimum | Maximum |
| --- | --- | --- | --- | --- | --- | --- | --- | --- |
|  |  |  |  |  | Lower Bound | Upper Bound |  |  |
| 4.00 | 3 | 0.088900 | 0.0034044 | 0.0019655 | 0.080443 | 0.097357 | 0.0854 | 0.0922 |
| 5.00 | 3 | 0.091100 | 0.0031193 | 0.0018009 | 0.083351 | 0.098849 | 0.0875 | 0.0930 |
| 6.00 | 3 | 0.087533 | 0.0006429 | 0.0003712 | 0.085936 | 0.089130 | 0.0868 | 0.0880 |
| Total | 9 | 0.089178 | 0.0028039 | 0.0009346 | 0.087022 | 0.091333 | 0.0854 | 0.0930 |

**Test of Homogeneity of Variances**

| Levene Statistic | df1 | df2 | Sig. |
| --- | --- | --- | --- |
| 2.318 | 2 | 6 | 0.180 |

**ANOVA**

|  | Sum of Squares | df | Mean Square | F | Sig. |
| --- | --- | --- | --- | --- | --- |
| Between Groups | 0.000 | 2 | 0.000 | 1.341 | 0.330 |
| Within Groups | 0.000 | 6 | 0.000 |  |  |
| Total | 0.000 | 8 |  |  |  |

Table S2: Determination of uric acid and creatinine in the artificial urine samples.

|  |  | Added (mM) | |  | Found (mM) | |  | % Recovery | |  | % RSD (n=3) | |
| --- | --- | --- | --- | --- | --- | --- | --- | --- | --- | --- | --- | --- |
|  |  | Uric acid | Creatinine |  | Uric acid | Creatinine |  | Uric acid | Creatinine |  | Uric acid | Creatinine |
| Sample-1 |  | 1.00 | 5.00 |  | 1.07 | 4.92 |  | 107 | 98 |  | 4.26 | 3.64 |
| Sample-2 |  | 2.00 | 20.00 |  | 2.07 | 20.46 |  | 104 | 102 |  | 3.66 | 2.93 |
| Sample-3 |  | 4.00 | 50.00 |  | 4.10 | 50.30 |  | 102 | 101 |  | 2.90 | 2.49 |
| Sample-4 |  | 6.00 | 100.00 |  | 6.29 | 102.87 |  | 105 | 103 |  | 3.18 | 3.11 |
| Sample-5 |  | 8.00 | 200.00 |  | 8.04 | 203.51 |  | 100 | 102 |  | 1.79 | 1.39 |

Table S3: Determination of uric acid to creatinine ratio in the control urine samples.

| Uric acid to creatinine ratios | |  | % Recovery |
| --- | --- | --- | --- |
| Added | Found |  |  |
| 0.65 | 0.64 |  | 98 |
| 0.75 | 0.75 |  | 100 |
| 0.85 | 0.86 |  | 101 |
| 0.95 | 0.94 |  | 99 |
